# Climate Change Anxiety Assessment: The Psychometric Properties of the Polish Version of the Climate Anxiety Scale

**DOI:** 10.3389/fpsyg.2022.870392

**Published:** 2022-05-11

**Authors:** Paweł Larionow, Michalina Sołtys, Paweł Izdebski, Karolina Mudło-Głagolska, Justyna Golonka, Maksym Demski, Maja Rosińska

**Affiliations:** Faculty of Psychology, Kazimierz Wielki University, Bydgoszcz, Poland

**Keywords:** assessment, climate anxiety, Climate Anxiety Scale, climate change, Polish validation, psychometric properties

## Abstract

The Climate Anxiety Scale (CAS) is a 13-item questionnaire for assessing climate anxiety (CA) as a psychological response to climate change. The CAS consists of two subscales, namely, cognitive impairment and functional impairment. This study aimed to validate the Polish version of the CAS. The sample included 603 respondents (344 females, 247 males, and 12 non-binary), aged 18–70 years (*M* = 25.32, *SD* = 9.59). Based on the exploratory factor analysis results, we proposed a 3-factor solution (i.e., intrusive symptoms, reflections on CA, and functional impairment), which seems to be theoretically more consistent with the content of the CAS statements. The confirmatory factor analysis showed that the original 2-factor solution and the 3-factor one had a satisfactory and a good fit to the data, respectively, as well as both were invariant across different gender, age, and educational level categories. Despite the fact that the 3-factor solution had the best-fit indices, we recommended to examine the CAS structure in different samples and use the overall CAS score in cross-cultural research. Cognitive and functional impairment subscales were positively correlated with personal experience of climate change, behavioral engagement, environmental identity, and environmental motives, but they were negatively correlated with climate change denial and sense of safety. The CAS subscales were correlated with depressive symptoms, but contrary to expectations, they were not associated with anxiety symptoms and any cognitive coping strategies. The Polish version of the CAS has satisfactory psychometric properties. Overall, we reported low CA levels in the Polish sample. Women and younger people experienced higher CA.

## Introduction

Climate anxiety (CA) and related terms such as climate distress and climate change anxiety define human negative emotions and states toward a global climate crisis and its threats (Clayton, 2020; Wu et al., 2020). Looking at the impact of climate change on psychological functioning, Thoma et al. (2021) described five pathways (i.e., biological, behavioral, cognitive, emotional, and social), the interaction of which can render an individual more or less susceptible to environmental stress factors, from the perspective of the vulnerability-stress model. Duggan et al. (2021) noted that experiencing climate change could cause a wide range of emotions, especially negative ones (i.e., anger, feeling exasperated, anxious, distressed, upset, or infuriated). Wu et al. (2020) postulated that a scientific priority in this area is (1) developing and validating reliable and accurate research tools for measuring CA, (2) determining the extent to which CA affects people's mental health, (3) identifying groups most affected by CA, and (4) promoting effective psychological support methods for people with high levels of CA. To implement these postulates in the Polish population, the first perspective research task is to conduct the Polish validation of the Climate Anxiety Scale (CAS), which was developed in the United States of America by Clayton and Karazsia (2020). It is important due to the fact that the predictors and psychological effects of CA, especially among young people who are the most likely to express anxiety about climate change, have been less explored so far.

The CAS is a 13-item self-report questionnaire for assessing climate change anxiety as a psychological response to climate change (Clayton and Karazsia, 2020). By developing the scale, Clayton and Karazsia (2020) conducted a series of exploratory factor analyses (EFA) on the pool of 22 items, which represented four factors: cognitive impairment, functional impairment, experience of climate change, and behavioral engagement. They considered that the cognitive impairment (eight items, e.g., *Thinking about climate change makes it difficult for me to concentrate*) and functional impairment (five items, e.g., *My concerns about climate change make it hard for me to have fun with my family or friends*) subscales consisting of a total of 13 statements form the CAS.

Despite the fact that the developed CAS scale showed satisfactory psychometric properties, it should be stressed that in the original study by Clayton and Karazsia (2020), the factor structure assessment of the 13-item CAS was not conducted. As for convergent and divergent validity, Clayton and Karazsia (2020) reported that the overall score of anxiety-depressive symptoms and negative emotionality was strongly associated with the cognitive and functional impairment subscales. However, the correlation between the CAS subscales and anxiety or depressive symptoms was not assessed separately. Clayton and Karazsia (2020) evidenced that the environmental identity was associated with cognitive impairment, and it was weakly correlated with functional impairment. Experience of climate change was positively associated with both the CAS subscales, but the behavioral engagement was not (Clayton and Karazsia, 2020).

Currently, only a few validation studies of the CAS have been conducted in different cultures. As for factor structure, a German validation study by Wullenkord et al. (2021) and an Italian validation study of the CAS conducted by Innocenti et al. (2021) did not confirm the 2-factor structure of the scale. The French validation study by Mouguiama-Daouda et al. (2022) showed the priority of the 2-factor over the 1-factor structure of the 13-item scale. However, model fit indices of the 2-factor model were close to the threshold limit value (Mouguiama-Daouda et al., 2022). The Philippine validation study by Simon et al. (2022) showed that the 2-factor model with four correlated errors had a good fit for the data, whereas the 2-factor one without correlated errors showed an inadequate fit in the sample of young Filipinos. Thus, these validation studies conducted in different cultures have revealed contradictory results in the CAS factor structure.

Regarding the convergent and divergent validity of the CAS, Wullenkord et al. (2021) reported that higher CA was associated with a higher level of the overall score on anxiety-depressive symptoms. Other studies have shown the presence of correlations of the CAS subscales with depressive symptoms or no correlations with anxiety symptoms (Innocenti et al., 2021; Mouguiama-Daouda et al., 2022). Therefore, the CAS subscales showed inconsistent correlations with depressive and anxiety symptoms. Wullenkord et al. (2021) evidenced that people with higher CA expressed less climate denial and stronger pro-environmental intentions as well as pro-environmental behaviors, which was reported by Innocenti et al. (2021). In contrast, there was no relationship between CA and behavioral engagement in the original study by Clayton and Karazsia (2020).

Summarizing the results of the CAS validation studies, it is necessary to clarify its factor structure and concurrent validity (i.e., the relationship of the CAS subscales with depressive and anxiety symptoms). Previous research has focused more on the psychological consequences of CA. However, possible predictors of CA have not been studied. As the CAS scale measures anxiety response to climate change, we assumed that cognitive coping strategies (as rather stable styles of dealing with negative life events) may be the correlates of CA, especially rumination, self-blame, catastrophizing, and lack of positive reappraisal, which are associated with the occurrence of anxiety and depressive symptoms (Martin and Dahlen, 2005; Garnefski and Kraaij, 2007).

The aim of this study was to validate the CAS in the Polish sample. Based on the above-described studies, we assumed that (1) the Polish version of the CAS is characterized by the 2-factor structure and is invariant across gender, education, and age categories; (2) CA is positively correlated with experiencing climate change, behavioral engagement, environmental identity, environmental concerns, depressive, and anxiety symptoms as well as is negatively associated with the level of climate change denial and sense of safety; (3) maladaptive cognitive coping strategies (catastrophizing, rumination, self-blame, and blaming others) are positively related to CA, whereas the adaptive ones (positive reappraisal, putting into perspective, positive refocusing, acceptance, and refocus on planning) are negatively related to CA.

## Materials and Methods

### Participants

The sample included 603 Polish adults (344 females, 247 males, and 12 non-binary) aged 18–70 years (*M* = 25.32, Me = 22.00, SD = 9.59). People with higher education made up 34.33% of respondents, whereas 65.67% had lower educational levels. Large cities (above 100,000 inhabitants) were home to 47.93% of the respondents, medium-sized towns (from 20,000 to 100,000) to 19.24%, small towns (up to 20,000) to 10.78%, and villages to 22.06%.

### Measures

1. The CAS by Clayton and Karazsia (2020) is a 13-item scale for assessing climate change anxiety. The CAS consists of two subscales, namely, the cognitive impairment subscale (eight items; e.g., *Thinking about climate change makes it difficult for me to sleep*) and the functional impairment subscale (five items; e.g., *I have problems balancing my concerns about sustainability with the needs of my family*). Respondents assessed how often the CAS statements are true of them using a 5-point Likert scale from 1 (*never*) to 5 (*almost always*). The subscales can be calculated independently, and an overall score can also be used.

2. The Experience of Climate Change Scale was developed by Clayton and Karazsia (2020) as a validation means for the CAS scale. The experience of climate change consists of three statements and measures an individual's perception of being affected by climate change (e.g., *I have been directly affected by climate change*). The responses are rated on a 5-point Likert scale from 1 (*strongly disagree*) to 5 (*strongly agree*).

3. The Behavioral Engagement Scale was designed by Clayton and Karazsia (2020) for the CAS scale validation. The scale consists of six statements related to the behavioral activity in the field of environmental care (e.g., *I recycle*; *I turn off lights*). The responses are rated on a 5-point Likert scale from 1 (*strongly disagree*) to 5 (*strongly agree*).

4. The Environmental Identity Scale-Revised (EID-R) version by Clayton et al. (2021) is a 14-item questionnaire developed for measuring the power of people's nature connectedness on both cognitive and emotional levels. Larionov (2020), who participated in the validation study of the EID scale, prepared a Polish translation of the revised EID. Respondents assess the statements (e.g., *I think of myself as a part of nature, not separate from it*) using a 7-point Likert scale from 1 (*not at all true of me*) to 7 (*completely true of me*).

5. The denial belief scale is a set of five separate statements developed by McCright and Dunlap (2011) to measure attitudes toward climate change denial. These statements (e.g., *Recent temperature increases are not primarily due to human activities*) were modified and combined into a one-factor scale. All modifications of the original version (modifications included altering “global warming” to “climate change”) were made with the permission of McCright and Dunlap (2011). The statements are assessed using a 5-point Likert scale ranging from 1 (*not at all true of me*) to 5 (*completely true of me*). In this study, we used the Polish version of the scale, which had previously been applied in cross-cultural research by Nartova-Bochaver et al. (in review) and showed high reliability.

6. The Environmental Motives Scale (EMS) by Schultz (2001) is a 12-item questionnaire for measuring concern with environmental issues rooted in a person's values. The EMS represents three types of environmental concern motives: focused on egoistic concerns (e.g., *my health*; *my future*), altruistic (e.g., *people in my country*; *children*), and biospheric (e.g., *animals*; *plants*). The responses are rated from 1 (*not important*) to 7 (*supreme importance*). The Polish translation of the EMS was prepared by one author of the manuscript. Preliminary studies indicated high reliability.

7. The Patient Health Questionnaire-4 (PHQ-4) by Kroenke et al. (2009) in its Polish version (Patient Health Questionnaire Screeners, 2022) was used. The PHQ-4 is a 4-item questionnaire for measuring anxiety and depressive symptoms in the previous 2 weeks, which uses a 4-point Likert scale from 0 (*not at all*) to 3 (*nearly every day*). The PHQ-4 has two subscales, namely, anxiety (two items, e.g., *Feeling nervous, anxious, or on edge*) and depression (two items, e.g., *Feeling down, depressed, or hopeless*). The overall score of anxiety-depressive symptoms can also be calculated.

8. The Sense of Safety subscale of the Safety Experience Questionnaire was developed in Poland by Klamut (2019) for evaluating the sense of safety level. The subscale has five statements (e.g., *I feel safe in the current reality*), which are evaluated on a 5-point Likert scale from 1 (*definitely not*) to 5 (*definitely yes*). The Polish version of the subscale is valid and reliable (Klamut, 2019).

9. The Cognitive Emotion Regulation Questionnaire (CERQ) by Garnefski et al. (2001) in Polish adaptation by Marszał-Wiśniewska and Fajkowska (2010) was used. The CERQ is a 36-item questionnaire for assessing nine cognitive coping strategies, among which there are adaptive (acceptance, positive refocusing, refocusing on planning, positive reappraisal, and putting into perspective) strategies, as well as maladaptive ones (self-blame, rumination, catastrophizing, and blaming others). The CERQ statements (e.g., *I keep thinking about how terrible it is what I have experienced*) are evaluated using a 5-point Likert scale from 1 [*(almost) never*] to 5 [*(almost) always*]. The Polish version of the CERQ is a valid and reliable tool (Marszał-Wiśniewska and Fajkowska, 2010).

In all the questionnaires, higher scores indicate higher levels of constructs being measured. In this study, all questionnaires showed high or satisfactory internal reliability (Cronbach's alpha; refer to Table 1).

**Table 1:** Descriptive statistics, Cronbach's alpha (α) coefficients, and gender differences.

**Scales**	**Total sample (females, males, non-binary)**				**Females**			**Males**			***p*-value (Mann–Whitney *U*-test)**
	***N***	**α**	***M***	***SD***	***N***	***M***	***SD***	***N***	***M***	***SD***	
Cognitive impairment	603	0.87	12.76	5.39	344	14.02	5.77	247	10.89	4.07	<0.001
Functional impairment	603	0.89	7.59	3.84	344	8.13	4.18	247	6.75	3.12	<0.001
Overall score of the CAS	603	0.92	20.34	8.68	344	22.16	9.37	247	17.64	6.67	<0.001
Experience of climate change	74	0.79	9.84	3.63	56	10.14	3.22	18	8.89	4.68	0.453
Behavioral engagement	74	0.68	25.34	3.71	56	25.84	3.10	18	23.78	4.98	0.149
Environmental identity	87	0.92	79.39	13.83	69	80.03	13.49	17	78.88	13.21	0.569
Biospheric concerns	64	0.85	22.81	4.73	50	23.28	4.61	14	21.14	4.97	0.108
Altruistic concerns	64	0.77	21.20	5.29	50	21.80	5.08	14	19.07	5.65	0.090
Egoistic concerns	64	0.75	22.58	4.59	50	22.84	4.64	14	21.64	4.48	0.291
Climate change denial	137	0.87	11.72	5.37	56	9.59	4.48	81	13.20	5.46	<0.001
Anxiety symptoms	106	0.88	2.25	1.79	50	2.88	1.87	55	1.67	1.54	0.001
Depressive symptoms	106	0.84	1.75	1.68	50	2.30	1.67	55	1.20	1.48	<0.001
Anxiety-depressive symptoms	106	0.89	4.01	3.21	50	5.18	3.23	55	2.87	2.77	<0.001
Sense of safety	106	0.85	19.25	3.42	50	19.24	2.98	55	19.36	3.73	0.564
Self-blame	64	0.83	11.20	3.53	50	11.06	3.68	14	11.71	3.00	0.511
Acceptance	64	0.62	13.58	2.92	50	14.06	2.58	14	11.86	3.46	0.045
Rumination	64	0.80	13.70	3.28	50	13.80	3.20	14	13.36	3.65	0.626
Positive refocusing	64	0.75	11.88	2.95	50	12.22	2.89	14	10.64	2.90	0.101
Refocus on planning	64	0.75	15.70	2.45	50	15.78	1.97	14	15.43	3.78	0.974
Positive reappraisal	64	0.80	14.64	3.26	50	14.74	3.17	14	14.29	3.65	0.685
Putting into perspective	64	0.84	12.80	3.75	50	13.14	3.80	14	11.57	3.41	0.194
Catastrophizing	64	0.67	8.97	3.03	50	9.16	2.87	14	8.29	3.60	0.252
Blaming others	64	0.86	9.64	3.31	50	9.88	3.17	14	8.79	3.77	0.131

### Translation Procedure

The translation procedure followed the recommendations of the International Test Commission. Four bilingual researchers translated the CAS, the Experience of Climate Change Scale, and the Behavioral Engagement Scale into Polish separately and then reached an agreement on the final translation. The Polish versions of the scales were translated back into English by a native speaker who speaks fluent Polish. The minor discrepancies were verified by bilingual experts.

### Research Procedure

This study was conducted online *via* Google Forms in the first part of 2021. The link to the survey was made available on social networking sites. This study was approved by the University Research Ethics Committee. All respondents provided their written informed consent before they answered the questions. There was no reimbursement for the participants. Not all respondents completed all the measures to avoid common method bias and stress during filling out the questionnaires.

### Statistical Analysis

Statistical analysis was carried out using IBM SPSS Statistics version 23 (for calculating descriptive statistics), and such statistical packages are the *lavaan* and *semTools* [for confirmatory factor analysis (CFA)], *EFAtools* and *psych* (for EFA and reliability analysis), *EFA.dimensions* [for Velicer's minimum average partial (MAP) test], and the *MVN* (for testing multivariate normality) using the R software version 4.1.0.

The EFA was conducted using the principal axis factoring with an Oblimin rotation. Parallel analysis and the visual scree test were used to determine the appropriate number of factors to retain. The result of Bartlett's test of sphericity and Kaiser–Meyer–Olkin (KMO) measure of sampling adequacy (with cutoff value > 0.70) was calculated (Lim and Jahng, 2019).

The following fit measures were taken into account for CFA: root mean square error of approximation (RMSEA), standardized root mean square residual (SRMR), comparative fit index (CFI), Tucker–Lewis index (TLI), and Akaike information criterion (AIC). RMSEA and SRMR values ≤ 0.08 indicate an acceptable fit. The CFI and TLI values ≥ 0.9 are acceptable (Hu and Bentler, 1999). The CAS factor models were compared using the AIC. A lower AIC value indicates a better fit (Byrne, 2013).

Measurement equivalence analysis was performed in configural, metric, and scalar levels across gender, age, and educational level groups. While testing metric and scalar invariance, the equivalence can be confirmed if the change in CFI is ≤ 0.01 and the one in RMSEA is ≤ 0.015 (Chen, 2007). We examined invariance in both young and older people. We divided our sample into two age groups of adults: one group aged under 25 years, i.e., 18–24 and the other group aged 25–55 years, based on the United Nations age classification [the age of young people is defined as 10–24 years (UNDESA, 2013)].

## Results

Table 1 presents descriptive statistics and gender differences (the Mann–Whitney *U*-test) for all the variables in this study. Women scored significantly higher than men in cognitive and functional impairment as well as the overall CAS score, anxiety, and depressive symptoms, as well as in acceptance. Men deny climate change significantly more than women.

Age was slightly negatively associated with the CAS scores (*N* = 603; functional impairment, *r*_*s*_ = −0.11, *p* = 0.006; cognitive impairment, *r*_*s*_ = −0.24, *p* < 0.001; overall CAS score, *r*_*s*_ = −0.20, *p* < 0.001). People with higher education (*N* = 207) scored significantly lower than people with lower educational levels (*N* = 396) in all the CAS scores (the Mann–Whitney *U*-test was used; functional impairment: Me = 5 vs. Me = 6.00, *p* = 0.003; cognitive impairment: Me = 9 vs. Me = 12.00, *p* < 0.001; overall CAS score: Me = 15 vs. Me = 18, *p* < 0.001).

### Factor Structure

**Figure 1:**
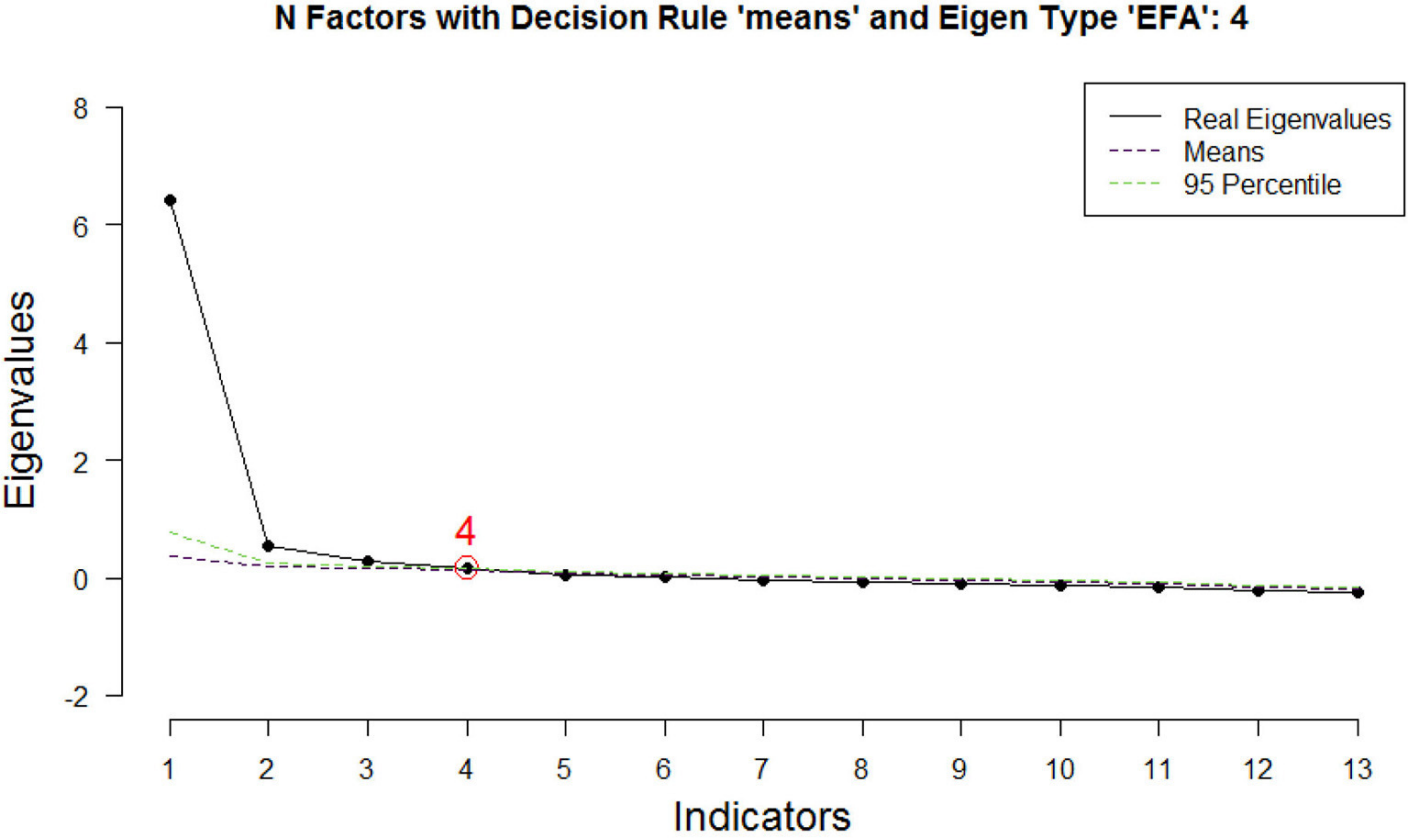
Scree plot of the 13-item EFA model.

The results of Bartlett's test of sphericity indicated that the correlation matrix was not random, X^2^_(78)_ = 4,552.26, *p* < 0.001, and the overall KMO value was 0.94 (meritorious). Following Velicer et al.'s recommendation (2000), which was cited by Watkins (2018), MAP and parallel analysis with the visual scree test were used to determine the appropriate number of factors to retain. Based on the polychoric correlations, the original MAP (Velicer, 1976) showed the retention of 2 factors, whereas the revised MAP (Velicer et al., 2000) identified the retention of 1 factor (refer to Supplementary Table 1). Parallel analysis, which was performed using 1,000 simulated random data sets, revealed that from 2 to 4 factors should be retained (refer to Figure 1).

Then, we conducted the EFA with the largest number of factors identified by the parallel analysis (i.e., 4). Later, we evaluated the 3-factor and the 2-factor solutions. The EFA, which was conducted using the principal axis factoring approach with Oblimin rotation, revealed that the 4-factor solution explained 60% of total variance; however, factor 4 was poorly loaded (0.34) only by item 7 (refer to Supplementary Table 2). Then, we researched the 3-factor solution, which explained 58% of the total variance and was theoretically more consistent with the content of the CAS statements (refer to Supplementary Table 3). Factor 1 (items 1–4) represents some difficulties, which are very similar to post-traumatic stress disorder symptoms (e.g., *I have nightmares about climate change*; *Thinking about climate change makes it difficult for me to concentrate*), thus, we called it *intrusive symptom* subscale. Factor 2 describes some reflections on respondents' reactions to climate change, entitled *reflections on climate anxiety* subscale (items 5–8; e.g., *I think, “why do I react to climate change this way?”*). Factor 3 corresponds with the original *functional impairment* subscale (items 9–13; e.g., *I have problems balancing my concerns about sustainability with the needs of my family*). Factor loadings ranged from 0.41 (for item 13) to 0.91 (for item 11), except for item 7 (*I write down my thoughts about climate change and analyze them*), which had low loadings (<0.27) on intended factors, which indicates a poor use of this CAS statement. As descriptive statistics (Table 2) evidenced, people's activity described by item 7 occurred very rarely in the Polish sample. Additionally, we noticed cross-loadings for item 13 (*My friends say I think about climate change too much*), which moderately loads intrusive symptoms and functional impairment. However, we decided to keep items 7 and 13 to maintain the CAS integrity for cross-cultural research.

**Table 2:** Descriptive statistics of the Climate Anxiety Scale (CAS) statements and standardized factor loadings from the confirmatory factor analysis (CFA) [robust maximum likelihood (ML) estimation, all *p* < 0.001].

**Statements**	***M(SD)***	**Skewness**	**Kurtosis**	**1-factor model**	**2-factor model**	**3-factor model**
1. Thinking about climate change makes it difficult for me to concentrate.	2.03 (1.10)	0.77	−0.26	0.748	0.783	0.783
2. Thinking about climate change makes it difficult for me to sleep.	1.49 (0.84)	1.81	3.04	0.786	0.804	0.833
3. I have nightmares about climate change.	1.35 (0.72)	2.21	4.46	0.569	0.603	0.607
4. I find myself crying because of climate change.	1.47 (0.90)	2.00	3.40	0.741	0.755	0.770
5. I think, “why can't I handle climate change better?”.	1.82 (1.13)	1.15	0.19	0.634	0.673	0.719
6. I go away by myself and think about why I feel this way about climate change.	1.80 (1.07)	1.23	0.67	0.598	0.646	0.766
7. I write down my thoughts about climate change and analyze them.	1.19 (0.57)	3.47	13.16	0.439	0.463	0.476
8. I think, “why do I react to climate change this way?”.	1.60 (0.97)	1.60	1.81	0.623	0.666	0.790
9. My concerns about climate change make it hard for me to have fun with my family or friends.	1.53 (0.93)	1.78	2.47	0.841	0.857	0.859
10. I have problems balancing my concerns about sustainability with the needs of my family.	1.75 (1.08)	1.33	0.81	0.679	0.691	0.690
11. My concerns about climate change interfere with my ability to get work or school assignments done.	1.41 (0.82)	2.20	4.63	0.835	0.882	0.882
12. My concerns about climate change undermine my ability to work to my potential.	1.43 (0.87)	2.20	4.36	0.825	0.864	0.862
13. My friends say I think about climate change too much.	1.46 (0.93)	2.09	3.50	0.667	0.656	0.658

**Figure 2:**
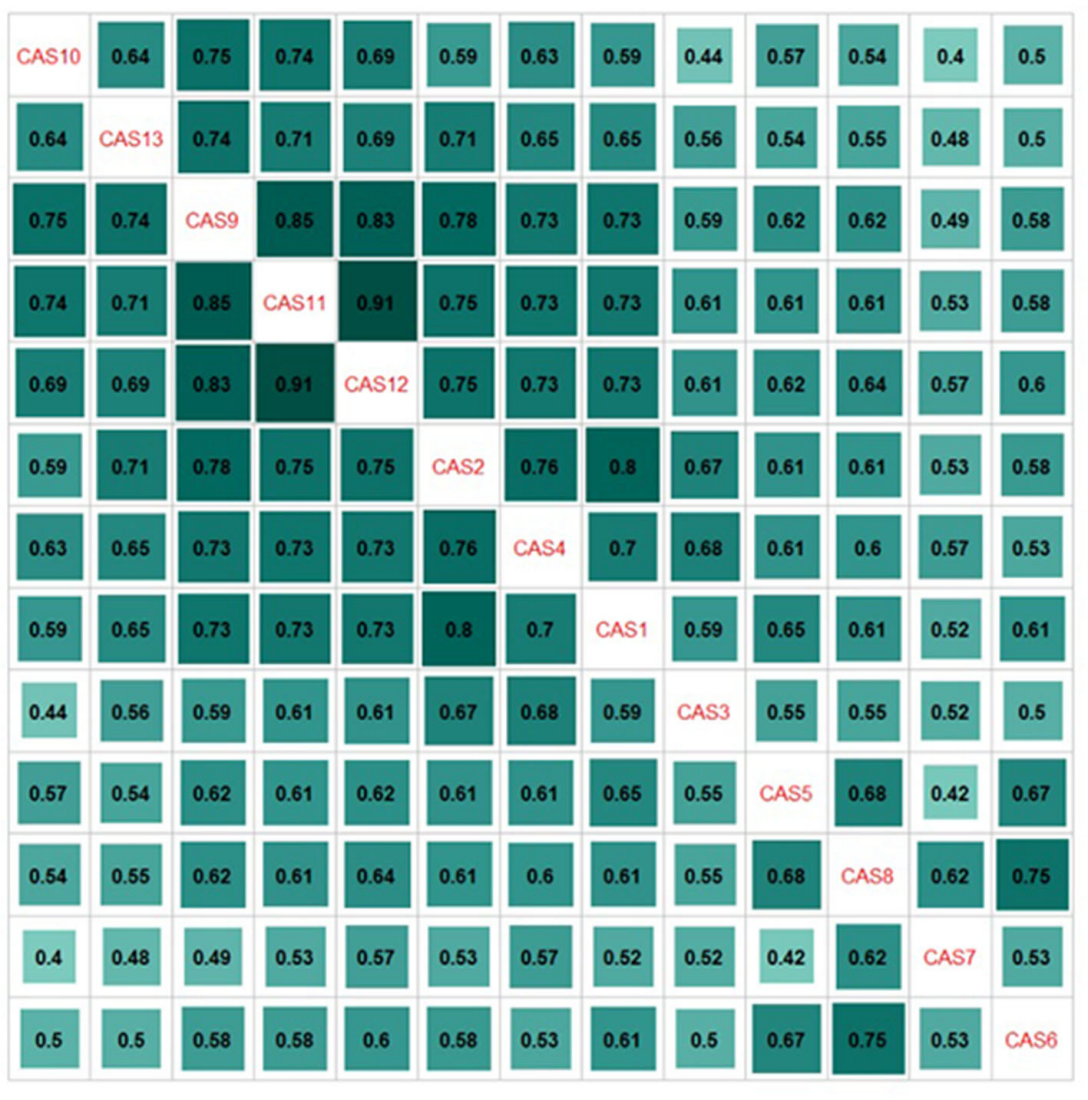
The polychoric correlation matrix for the CAS items (*N* = 603).

Then, we researched the 2-factor EFA solution, which explained approximately 55% of the total variance (refer to Supplementary Table 4). Factor 1 (items 1–4 and 9–13) represented the *intrusive symptoms* subscale and the original *functional impairment* subscale, whereas factor 2 represented the *reflections on climate anxiety* subscale (items 5–8). We could not compare our EFA results with the original ones because Clayton and Karazsia (2020) had not presented them. We also provided the polychoric correlations between the CAS items (refer to Figure 2). Summarizing the EFA results, we suggested that the 3-factor Polish model is the most appropriate solution empirically and theoretically.

The CFA was performed to assess fit indices of the 1-factor, original 2-factor, and 3-factor solutions. The Henze–Zirkler's multivariate normality test indicated the absence of multivariate normality of the CAS items (HZ = 52.88, *p* < 0.001). Due to this, robust maximum likelihood (robust ML) estimation was applied. The description of models and their goodness-of-fit indices are presented in Table 3. Factor loadings (all *p* < 0.001) for the items within all the examined models are displayed in Table 2.

**Table 3:** Goodness-of-fit indices for the CAS models (robust ML estimation).

**Models**	***χ^2^/df***	**CFI**	**TLI**	**RMSEA (90% CI)**	**SRMR**	**AIC**
1-factor model (items 1–13)	300.24/65 = 4.62	0.892	0.870	0.110 (0.098; 0.123)	0.057	16,694.551
2-factor correlated model: cognitive impairment (items 1–8) and functional impairment (items 9–13)	205.33/64 = 3.21	0.936	0.922	0.085 (0.072; 0.099)	0.047	16,496.321
3-factor correlated model: intrusive symptoms (items 1–4), reflections on CA (items 5–8) and functional impairment (items 9–13)	135.078/62 = 2.18	0.968	0.959	0.062 (0.048; 0.076)	0.040	16,355.976

The 1-factor model showed a poor fit to the data. The original 2-factor model provided a satisfactory one, but RMSEA was unacceptable. The estimated covariance between the cognitive impairment and the functional impairment subscales was 0.866 (*p* < 0.001). The 3-factor model (intrusive symptoms, reflections on CA, and functional impairment) reflects our 3-factor Polish solution. The estimated covariances in the 3-factor model were as follows: 0.790 between intrusive symptoms and reflections on CA, 0.873 between intrusive symptoms and functional impairment, and 0.716 between reflections on CA and functional impairment (all *p* < 0.001). The 3-factor model provided a good fit according to all fit indices. Thus, the original 2-factor and 3-factor models are the most appropriate solutions. However, there is still good support for using the overall CAS score, considering strong correlations between the subscales in the models and the fact that the overall score has high reliability.

We conducted a series of invariance analyses with respect to configural, metric, and scalar invariance across gender groups [females (*N* = 344) vs. males (*N* = 247)], two age groups [18–24 (*N* = 397) vs. 25–55 years old (*N* = 194)], and two educational level categories [higher education (*N* = 207) vs. lower than higher education (*N* = 396)]. In general, the analyses showed that both the 2-factor and the 3-factor models were invariant regarding their configural, metric, and scalar invariance across different gender, age, and educational level categories (ΔCFI <0.01 and ΔRMSEA <0.015) (refer to Supplementary Table 5). Only in the case of metric invariance across gender groups, the ΔCFI was only slightly higher than |0.01| (i.e., −0.011 and −0.012 for the 2-factor and the 3-factor models, respectively). However, the ΔRMSEA was < |0.015|, thus, it could be considered that metric invariance across genders was supported for the 2-factor and the 3-factor models.

### Convergent and Divergent Validity

Cognitive and functional impairment subscales were positively correlated with the experience of climate change, behavioral engagement, environmental identity, and environmental motives (except correlation between functional impairment and altruistic motives). In contrast, they were negatively correlated with climate change denial and sense of safety. The CAS subscales were positively related to depressive symptoms but surprisingly were not associated with anxiety symptoms or any cognitive coping strategies (refer to Supplementary Table 6).

### Internal Consistency Reliability

McDonald's omega total (ω_t_) and omega hierarchical (ω_h_) values indicated high reliability of the overall score and satisfactory subscale reliability (refer to Reise et al., 2013) in the whole sample (*N* = 603) in the generic model estimated using the Schmid Leiman Transformation. The overall CAS score was characterized by ω_t_ = 0.94, whereas ω_h_ was 0.89 and 0.87 in the 2-factor model and the 3-factor one, respectively. In the 2-factor model, omega values were as follows: cognitive impairment (ω_t_ = 0.93, ω_h__=_ 0.90) and functional impairment (ω_t_ = 0.80, ω_h_ = 0.52). In the 3-factor model, omega values were as follows: intrusive symptoms (ω_t_ = 0.89, ω_h_ = 0.72), reflections on CA (ω_t_= 0.80, ω_h_ = 0.53), and functional impairment (ω_t_ = 0.85; ω_h_ = 0.83). Cronbach's alpha (α) was high for all subscales of the 2-factor and 3-factor solutions (overall CAS score α = 0.92, cognitive impairment α = 0.87, functional impairment α = 0.89, intrusive symptoms α = 0.83, and reflections on CA α = 0.77) in the whole sample (*N* = 603).

## Discussion

The Polish validation study of the CAS showed that both the original 2-factor solution and the 3-factor Polish one have a satisfactory and a good fit to the data, respectively. Moreover, both are invariant across different gender, age, and educational level categories. The 3-factor model consists of *intrusive symptoms* subscale (this is factor 1, which reflects difficulties with concentration, sleep, crying, and the presence of nightmares due to climate change; refer to items 1–4), *reflections on climate anxiety* subscale (factor 2; it represents the analysis of thoughts and feelings about climate change; refer to items 5–8), and the *functional impairment* subscale of the original CAS (factor 3; refer to items 9–13). Despite the fact that the 3-factor model seems to be theoretically more consistent with the content of the CAS statements and it has the best-fit indices, we recommend to use the overall CAS score in cross-cultural research. However, we do not exclude that future validation studies in different cultures will confirm the 3-factor solution. Exploratory factor analysis studies of the CAS are required to examine its factor structure in different cultures.

As for concurrent validity, most of our results support previous findings on the CA correlates, i.e., the experience of climate change, environmental identity, and depressive symptoms (Clayton and Karazsia, 2020; Mouguiama-Daouda et al., 2022) as well as climate denial (Wullenkord et al., 2021). We also evidenced that biospheric concerns have a higher positive correlation with CA than egoistic and altruistic ones. Thus, the person's aspiration to protect the wildlife is associated with CA more than the aspiration to take care of themselves and the future generations.

Contrary to our hypothesis, all CAS subscales and the overall score were not correlated with cognitive coping strategies. Surprisingly, the cognitive impairment subscale and the overall CAS score were correlated with depressive symptoms, whereas there was no relationship between CAS subscales and anxiety symptoms measured separately. Similar results were obtained in the study by Mouguiama-Daouda et al. (2022). In compliance with the results by Wullenkord et al. (2021), in our study, the overall score of anxiety-depressive symptoms was positively associated with CA. All subscales and the overall CAS score were correlated with a lower sense of safety. Therefore, our results support previous findings concerning contradictory associations of CA with anxiety and depressive symptoms. We agree with Wullenkord et al. (2021, p. 1) that the CAS appears “*to measure a general climate-related emotional impairment, rather than distinctly and comprehensively capturing climate anxiety*.” Nevertheless, our results also support Clayton and Karazsia (2020) thesis that climate change anxiety is a complex psychological response associated with negative emotions (e.g., Ojala, 2007; Clayton, 2020). In our opinion, the CAS seems to measure the emotional and cognitive response (not unequivocally maladaptive) related to climate change. It should be stressed that some CAS statements seem to reflect the significance of the climate change problem for an individual and possibly the willingness to solve it rather than difficulties or impairment [e.g., *I write down my thoughts about climate change and analyze them* (item 7)]. A one-time measurement does not allow to draw a conclusion whether the complex response to CA is maladaptive or adaptive. For that reason, longitudinal research is recommended.

Experiencing climate change can have different effects on psychological functioning in different groups (Clayton, 2020). In our study, women and younger individuals revealed significantly higher CA. Our results are consistent with previous findings (Clayton and Karazsia, 2020; Wullenkord et al., 2021). Our results on socio-demographic differences in CA are preliminary; therefore, further research on them, taking into account other significant variables (e.g., religion) and possible moderation effects, is required. Schwartz et al. (2022) noted the role of environmental activism as a buffer in the relationship between CA and mental health. They stressed that it was collective climate activism but not an individual one, which was related to lower negative CA effects on depressive symptoms. In this regard, we consider it promising to identify the groups of individuals with different CA levels as well as with different behavioral engagements in climate change mitigation in a broad general sample. The person-oriented approach by latent profile analysis can be used for that. This would make it possible to describe risk groups and provide them with psychological support program development, as well as with social and political programs targeted at certain groups that are hardly interested in climate change or deny it. Additionally, we believe that studying CA predictors is an important research area to find intervention targets in people with high levels of CA.

## Limitations

The validation study was conducted in a broad general sample with a wide range of ages and an almost equal number of men and women. However, in our sample, young people predominated, and the empirical distribution of most variables was deviating from normal distribution; therefore, the possibility of generalizing the results is limited.

This is a cross-sectional study. No conclusion can be drawn regarding the temporal order of CA and its correlates. The test–retest reliability was not assessed.

## Data Availability Statement

The raw data supporting the conclusions of this article will be made available by the authors, without undue reservation.

## Ethics Statement

The studies involving human participants were reviewed and approved by Research Ethics Committee in Kazimierz Wielki University. The patients/participants provided their written informed consent to participate in this study.

## Author Contributions

PL: conceptualization, formal analysis, data curation, investigation, methodology, writing—review and editing, supervision, and project administration. MS: conceptualization, formal analysis, data curation, investigation, methodology, and writing—review and editing. PI: conceptualization, data curation, and methodology. KM-G: data curation, investigation, and methodology. JG, MD, and MR: data curation and methodology. All authors approved the final manuscript and agreed to the authorship order.

## Conflict of Interest

The authors declare that the research was conducted in the absence of any commercial or financial relationships that could be construed as a potential conflict of interest.

## Publisher's Note

All claims expressed in this article are solely those of the authors and do not necessarily represent those of their affiliated organizations, or those of the publisher, the editors and the reviewers. Any product that may be evaluated in this article, or claim that may be made by its manufacturer, is not guaranteed or endorsed by the publisher.

## References

[B1] Byrne B. M (2013). Structural Equation Modeling With AMOS: Basic Concepts, Applications, and Programming, 2nd Edn. New York, NY: Routledge.

[B2] Chen F. F (2007). Sensitivity of goodness of fit indexes to lack of measurement invariance. Struct. Eq. Model. 14, 464–504. 10.1080/10705510701301834

[B3] Clayton S (2020). Climate anxiety: psychological responses to climate change. J. Anxiety Disord. 74, 102263. 10.1016/j.janxdis.2020.102263 32623280

[B4] Clayton S. Czellar S. Nartova-Bochaver S. Skibins J. C. Salazar G. Tseng Y.-C.. (2021). Cross-cultural validation of a revised environmental identity scale. Sustainability 13, 2387. 10.3390/su13042387

[B5] Clayton S. Karazsia B. T. (2020). Development and validation of a measure of climate change anxiety. J. Environ. Psychol. 69, 101434. 10.1016/j.jenvp.2020.101434

[B6] Duggan J. Haddaway N. R. Badullovich N. (2021). Climate emotions: it is ok to feel the way you do. Lancet Planet. Health 5, e854–e855. 10.1016/S2542-5196(21)00318-1 34895490

[B7] Garnefski N. Kraaij V. (2007). The cognitive emotion regulation questionnaire: psychometric features and prospective relationships with depression and anxiety in adults. Eur. J. Psychol. Assess. 23, 141–149. 10.1027/1015-5759.23.3.141

[B8] Garnefski N. Kraaij V. Spinhoven P. (2001). Negative life events, cognitive emotion regulation and emotional problems. Pers. Individ. Differ. 30, 1311–1327. 10.1016/S0191-8869(00)00113-616791542

[B9] Hu L. Bentler P. M. (1999). Cutoff criteria for fit indexes in covariance structure analysis: conventional criteria versus new alternatives. Struct. Eq. Model. 6, 1–55. 10.1080/10705519909540118

[B10] Innocenti M. Santarelli G. Faggi V. Castellini G. Manelli I. Magrini G.. (2021). Psychometric properties of the italian version of the climate change anxiety scale. J. Clim. Change Health 3, 100080. 10.1016/j.joclim.2021.100080

[B11] Klamut R (2019). Two-factor model of safety experience – theoretical assumptions and empirical verification: safety experience questionnaire. Pol. Forum Psychol. 24, 308–332. 10.14656/PFP20190303

[B12] Kroenke K. Spitzer R. L. Williams J. B. W. Lowe B. (2009). An ultra-brief screening scale for anxiety and depression: the PHQ-4. Psychosomatics 50, 613–621. 10.1176/appi.psy.50.6.613 19996233

[B13] Larionov P (2020). Toward an environmental psychology: psychometric properties of the Polish version of the Environmental Identity Scale. Q. J. Fides Et Ratio 44, 190–208. 10.34766/fetr.v44i4.452

[B14] Lim S. Jahng S. (2019). Determining the number of factors using parallel analysis and its recent variants. Psychol. Methods 24, 452–467. 10.1037/met0000230 31180694

[B15] Marszał-Wiśniewska M. Fajkowska M. (2010). Psychometic properties of the Cognitive Emotion Regulation Questionnaire (CERQ): results of the studies on the Polish sample. Stud. Psychol. 49, 19–39.

[B16] Martin R. C. Dahlen E. R. (2005). Cognitive emotion regulation in the prediction of depression, anxiety, stress, and anger. Pers. Individ. Differ. 39, 1249–1260. 10.1016/j.paid.2005.06.004

[B17] McCright A. M. Dunlap R. E. (2011). Cool dudes: the denial of climate change among conservative white males in the United States. Glob. Environ. Change 21, 1163–1172. 10.1016/j.gloenvcha.2011.06.003

[B18] Mouguiama-Daouda C. Blanchard M. A. Coussement C. Heeren A. (2022). On the measurement of climate change anxiety: French validation of the climate anxiety scale. Psychologica Belgica 62, 123–135. 10.5334/pb.1137 35414943 PMC8954884

[B19] Nartova-Bochaver S. K. Donat M. Kiral Ucar G. Korneev A. Heidmets M. Kamble S. (in review). The role of environmental identity individualism/collectivism in predicting climate change denial: evidence from nine countries. J. Environ. Psychol. .

[B20] Ojala M (2007). Hope and Worry: Exploring Young People's Values, Emotions, and Behavior Regarding Global Environmental Problems (doctoral Dissertation). Orebro: Orebro University.

[B21] Patient Health Questionnaire Screeners (2022). Available online at: https://www.phqscreeners.com/ (accessed March 15, 2022).

[B22] Reise S. Bonifay W. Haviland M. (2013). Scoring and modeling psychological measures in the presence of multidimensionality. J. Person. Assess. 95, 129–140, 10.1080/00223891.2012.725437 23030794

[B23] Schultz W. P (2001). The structure of environmental concern: concern for self, other people, and the biosphere. J. Environ. Psychol. 21, 327–339. 10.1006/jevp.2001.0227

[B24] Schwartz S. E. O. Benoit L. Clayton S. Parnes M. F. Swenson L. Lowe S. R. (2022). Climate change anxiety and mental health: Environmental activism as buffer. Curr. Psychol. 10.1007/s12144-022-02735-6 PMC8883014 35250241

[B25] Simon P. D. Pakingan K. A. Aruta J. J. B. R. (2022). Measurement of climate change anxiety and its mediating effect between experience of climate change and mitigation actions of Filipino youth. Educ. Dev. Psychol. 39, 17–27. 10.1080/20590776.2022.2037390

[B26] Thoma M. V. Rohleder N. Rohner S. L. (2021). Clinical ecopsychology: the mental health impacts and underlying pathways of the climate and environmental crisis. Front. Psychiatry 12, 675936. 10.3389/fpsyt.2021.675936 34093283 PMC8175799

[B27] UNDESA (2013). Definition of Youth. United Nations Department of Economic and Social Affairs. Available online at: https://www.un.org/esa/socdev/documents/youth/fact-sheets/youth-definition.pdf (accessed January 31, 2022).

[B28] Velicer W. F (1976). Determining the number of components from the matrix of partial correlations. Psychometrika 41, 321–327. 10.1007/BF02293557

[B29] Velicer W. F. Eaton C. A. Fava J. L. (2000). “Construct explication through factor or component analysis: a review and evaluation of alternative procedures for determining the number of factors or components,” in Problems and Solutions in Human Assessment, eds R. D. Goffin, and E. Helmes (Boston, MA: Springer), 41–71.

[B30] Watkins M. W (2018). Exploratory factor analysis: a guide to best practice. J. Black Psychol. 44, 219–246. 10.1177/0095798418771807

[B31] Wu J. Snell G. Samji H. (2020). Climate anxiety in young people: a call to action. Lancet Planet. Health 4, e435–e436. 10.1016/S2542-5196(20)30223-0 32918865

[B32] Wullenkord M. C. Tröger J. Hamann K. R. S. Loy L. S. Reese G. (2021). Anxiety and climate change: a validation of the Climate Anxiety Scale in a German-speaking quota sample and an investigation of psychological correlates. Clim. Change 168, 1–23. 10.1007/s10584-021-03234-6

