# Supplementary material for: Climate Change Anxiety Assessment: The Psychometric Properties of the Polish Version of the Climate Anxiety Scale
Source: Front Psychol. 2022 May 11;13:870392. doi: 10.3389/fpsyg.2022.870392 (PMC9130850; doi:10.3389/fpsyg.2022.870392)
Supplement: Supplementary file 1 [file Table_1.docx]

**Supplementary Table 1.** The MAP test results for different numbers of components.

| **Number of components** | **Average squared correlation** | **Average 4rth power correlation** |
| --- | --- | --- |
| 0 | 0.40462 | 0.18020 |
| 1 | 0.03700 | **0.00317** |
| 2 | **0.03389** | 0.00335 |
| 3 | 0.04244 | 0.00630 |
| 4 | 0.05669 | 0.01346 |
| 5 | 0.07453 | 0.02217 |
| 6 | 0.10045 | 0.03349 |
| 7 | 0.12791 | 0.04406 |
| 8 | 0.16474 | 0.07750 |
| 9 | 0.26413 | 0.14833 |
| 10 | 0.36497 | 0.24395 |
| 11 | 0.58773 | 0.47182 |
| 12 | 1.00000 | 1.00000 |

*Note*. The smallest average squared correlation is 0.03389. The smallest average 4rth power correlation is 0.00317. These indices of correlations are shown in bold.
