# Supplementary material for: Climate Change Anxiety Assessment: The Psychometric Properties of the Polish Version of the Climate Anxiety Scale
Source: Front Psychol. 2022 May 11;13:870392. doi: 10.3389/fpsyg.2022.870392 (PMC9130850; doi:10.3389/fpsyg.2022.870392)
Supplement: Supplementary file 2 [file Table_2.DOCX]

**Supplementary Table 2.** Results of EFA with the fixed 4 factors by principal axis estimation method with an Oblimin rotation (*N* = 603).

| **Items** | **Rotated factor loadings** | | | |
| --- | --- | --- | --- | --- |
|  | **Factor 1** | **Factor 2** | **Factor 3** | **Factor 4** |
| 1. Thinking about climate change makes it difficult for me to concentrate. | 0.098 | **0.485** | 0.272 | -0.141 |
| 2. Thinking about climate change makes it difficult for me to sleep. | 0.084 | **0.776** | 0.006 | -0.107 |
| 3. I have nightmares about climate change. | -0.064 | **0.654** | 0.045 | 0.107 |
| 4. I find myself crying because of climate change. | 0.170 | **0.640** | 0.002 | 0.065 |
| 5. I think, “why can’t I handle climate change better?”. | 0.028 | 0.157 | **0.602** | -0.181 |
| 6. I go away by myself and think about why I feel this way about climate change. | 0.013 | -0.031 | **0.798** | 0.012 |
| 7. I write down my thoughts about climate change and analyze them. | 0.096 | 0.220 | 0.194 | **0.342** |
| 8. I think, “why do I react to climate change this way?”. | 0.049 | -0.011 | **0.769** | 0.150 |
| 9. My concerns about climate change make it hard for me to have fun with my family or friends. | **0.664** | 0.200 | 0.044 | -0.142 |
| 10. I have problems balancing my concerns about sustainability with the needs of my family. | **0.580** | -0.001 | 0.167 | -0.159 |
| 11. My concerns about climate change interfere with my ability to get work or school assignments done. | **0.939** | -0.017 | -0.031 | 0.085 |
| 12. My concerns about climate change undermine my ability to work to my potential. | **0.826** | 0.028 | 0.026 | 0.119 |
| 13. My friends say I think about climate change too much. | **0.322** | **0.402** | -0.007 | -0.078 |
| Proportion of total variance | 0.501 | 0.054 | 0.028 | 0.021 |

*Note.* Factor loadings > 0.30 are shown in bold.
