# Supplementary material for: Climate Change Anxiety Assessment: The Psychometric Properties of the Polish Version of the Climate Anxiety Scale
Source: Front Psychol. 2022 May 11;13:870392. doi: 10.3389/fpsyg.2022.870392 (PMC9130850; doi:10.3389/fpsyg.2022.870392)
Supplement: Supplementary file 3 [file Table_3.docx]

**Supplementary Table 3.** Results of EFA with the fixed 3 factors by principal axis estimation method with an Oblimin rotation (*N* = 603).

| **Items** | **Rotated factor loadings** | | |
| --- | --- | --- | --- |
|  | **Factor 1** | **Factor 2** | **Factor 3** |
| 1. Thinking about climate change makes it difficult for me to concentrate. | **0.402** | 0.237 | 0.211 |
| 2. Thinking about climate change makes it difficult for me to sleep. | **0.678** | -0.006 | 0.200 |
| 3. I have nightmares about climate change. | **0.653** | 0.079 | -0.076 |
| 4. I find myself crying because of climate change. | **0.616** | 0.018 | 0.192 |
| 5. I think, “why can’t I handle climate change better?”. | 0.120 | **0.521** | 0.129 |
| 6. I go away by myself and think about why I feel this way about climate change. | -0.051 | **0.826** | 0.012 |
| 7. I write down my thoughts about climate change and analyze them. | 0.242 | 0.268 | 0.013 |
| 8. I think, “why do I react to climate change this way?”. | 0.022 | **0.787** | 0.004 |
| 9. My concerns about climate change make it hard for me to have fun with my family or friends. | 0.120 | -0.003 | **0.775** |
| 10. I have problems balancing my concerns about sustainability with the needs of my family. | -0.064 | 0.110 | **0.678** |
| 11. My concerns about climate change interfere with my ability to get work or school assignments done. | -0.013 | -0.017 | **0.907** |
| 12. My concerns about climate change undermine my ability to work to my potential. | 0.039 | 0.048 | **0.784** |
| 13. My friends say I think about climate change too much. | **0.337** | -0.030 | **0.406** |
| Proportion of total variance | 0.027 | 0.053 | 0.500 |

*Note.* Factor loadings > 0.30 are shown in bold.
