# Supplementary material for: Climate Change Anxiety Assessment: The Psychometric Properties of the Polish Version of the Climate Anxiety Scale
Source: Front Psychol. 2022 May 11;13:870392. doi: 10.3389/fpsyg.2022.870392 (PMC9130850; doi:10.3389/fpsyg.2022.870392)
Supplement: Supplementary file 4 [file Table_4.docx]

**Supplementary Table 4.** Results of EFA with the fixed 2 factors by principal axis estimation method with an Oblimin rotation (*N* = 603).

| **Items** | **Rotated factor loadings** | |
| --- | --- | --- |
|  | **Factor 1** | **Factor 2** |
| 1. Thinking about climate change makes it difficult for me to concentrate. | **0.500** | **0.313** |
| 2. Thinking about climate change makes it difficult for me to sleep. | **0.686** | 0.134 |
| 3. I have nightmares about climate change. | **0.384** | 0.235 |
| 4. I find myself crying because of climate change. | **0.635** | 0.145 |
| 5. I think, “why can’t I handle climate change better?”. | 0.191 | **0.554** |
| 6. I go away by myself and think about why I feel this way about climate change. | -0.051 | **0.812** |
| 7. I write down my thoughts about climate change and analyze them. | 0.171 | **0.334** |
| 8. I think, “why do I react to climate change this way?”. | -0.027 | **0.819** |
| 9. My concerns about climate change make it hard for me to have fun with my family or friends. | **0.911** | -0.070 |
| 10. I have problems balancing my concerns about sustainability with the needs of my family. | **0.661** | 0.023 |
| 11. My concerns about climate change interfere with my ability to get work or school assignments done. | **0.932** | -0.109 |
| 12. My concerns about climate change undermine my ability to work to my potential. | **0.849** | -0.028 |
| 13. My friends say I think about climate change too much. | **0.680** | -0.004 |
| Proportion of total variance | 0.497 | 0.051 |

*Note.* Factor loadings > 0.30 are shown in bold.
