# Supplementary material for: Climate Change Anxiety Assessment: The Psychometric Properties of the Polish Version of the Climate Anxiety Scale
Source: Front Psychol. 2022 May 11;13:870392. doi: 10.3389/fpsyg.2022.870392 (PMC9130850; doi:10.3389/fpsyg.2022.870392)
Supplement: Supplementary file 5 [file Table_5.DOCX]

**Supplementary Table 5**. Goodness-of-fit indices for invariance analysis of the CAS in terms of socio-demographic variables.

| **Variable 1: gender (females and males)** | | | | | | | | | | | |
| --- | --- | --- | --- | --- | --- | --- | --- | --- | --- | --- | --- |
| **Level of invariance** | **2-factor correlated model** | | | | | | **3-factor correlated model** | | | | |
|  | **χ^2^(df)** | **CFI** | **ΔCFI** | **RMSEA** | **ΔRMSEA** | | **χ^2^(df)** | **CFI** | **ΔCFI** | **RMSEA** | **ΔRMSEA** |
| Configural | 579.71 (128) | 0.920 | — | 0.093 | — | | 401.46 (124) | 0.960 | — | 0.067 | — |
| Metric | 653.75 (139) | 0.909 | -0.011 | 0.095 | 0.002 | | 476.71 (134) | 0.948 | -0.012 | 0.073 | 0.006 |
| Scalar | 700.04 (150) | 0.900 | -0.009 | 0.096 | 0.001 | | 519.91 (144) | 0.939 | -0.009 | 0.076 | 0.003 |
| **Variable 2: age (18–24 and 25–55 years old)** | | | | | | | | | | | |
| **Level of invariance** | **2-factor correlated model** | | | | | | **3-factor correlated model** | | | | |
|  | **χ^2^(df)** | **CFI** | **ΔCFI** | **RMSEA** | **ΔRMSEA** | | **χ^2^(df)** | **CFI** | **ΔCFI** | **RMSEA** | **ΔRMSEA** |
| Configural | 599.19 (128) | 0.915 | — | 0.097 | — | | 450.34 (124) | 0.949 | — | 0.076 | — |
| Metric | 632.46 (139) | 0.914 | -0.001 | 0.094 | -0.003 | | 480.64 (134) | 0.948 | -0.001 | 0.074 | -0.002 |
| Scalar | 659.79 (150) | 0.909 | -0.005 | 0.092 | -0.002 | | 514.15 (144) | 0.941 | -0.007 | 0.076 | 0.002 |
| **Variable 3: Educational level (higher educational level and lower levels)** | | | | | | | | | | | |
| **Level of invariance** | **2-factor correlated model** | | | | | **3-factor correlated model** | | | | | |
|  | **χ^2^(df)** | **CFI** | **ΔCFI** | **RMSEA** | **ΔRMSEA** | **χ^2^(df)** | | **CFI** | **ΔCFI** | **RMSEA** | **ΔRMSEA** |
| Configural | 576.18 (128) | 0.928 | — | 0.089 | — | 431.39 (124) | | 0.959 | — | 0.068 | — |
| Metric | 600.10 (139) | 0.930 | 0.002 | 0.085 | -0.004 | 451.20 (134) | | 0.961 | 0.002 | 0.064 | -0.004 |
| Scalar | 611.31 (150) | 0.930 | 0 | 0.082 | -0.003 | 464.39 (144) | | 0.960 | -0.001 | 0.062 | -0.002 |
