# Supplementary material for: Climate Change Anxiety Assessment: The Psychometric Properties of the Polish Version of the Climate Anxiety Scale
Source: Front Psychol. 2022 May 11;13:870392. doi: 10.3389/fpsyg.2022.870392 (PMC9130850; doi:10.3389/fpsyg.2022.870392)
Supplement: Supplementary file 6 [file Table_6.docx]

**Supplementary Table 6**. Spearman's correlations between the CAS subscales and the analyzed variables.

|  |  | **Original 2-factor solution** | | **Polish 3-factor solution** | | |
| --- | --- | --- | --- | --- | --- | --- |
| **Scales** | **Overall CAS score** | **Cognitive impairment subscale (factor 1; 1–8 items)** | **Functional impairment subscale (factor 2; 9–13 items)** | **Intrusive symptoms subscale (factor 1; items 1–4)** | **Reflections on climate anxiety subscale (factor 2; 5–8 items)** | **Functional impairment subscale (factor 3; 9–13 items)** |
| Experience of climate change (*N* = 74) | 0.44*** | 0.38*** | 0.50*** | 0.45*** | 0.26* | 0.50*** |
| Behavioral engagement (*N* = 74) | 0.45*** | 0.42*** | 0.43*** | 0.40*** | 0.34** | 0.43*** |
| Environmental identity (*N* = 87) | 0.45*** | 0.43*** | 0.35*** | 0.49*** | 0.28** | 0.35*** |
| Biospheric concerns (*N* = 64) | 0.46*** | 0.38** | 0.43*** | 0.46*** | 0.28* | 0.43*** |
| Altruistic concerns (*N* = 64) | 0.30* | 0.28* | 0.22 | 0.34** | 0.24 | 0.22 |
| Egoistic concerns (*N* = 64) | 0.36** | 0.31* | 0.34** | 0.37** | 0.24 | 0.34** |
| Climate change denial (*N* = 137) | -0.65*** | -0.62*** | -0.55*** | -0.57*** | -0.50*** | -0.55*** |
| Anxiety symptoms (*N* = 106) | 0.12 | 0.13 | 0.03 | 0.10 | 0.11 | 0.03 |
| Depressive symptoms (*N* = 106) | 0.26** | 0.26** | 0.14 | 0.27** | 0.20* | 0.14 |
| Anxiety-depressive symptoms (*N* = 106) | 0.21* | 0.22* | 0.10 | 0.20* | 0.17 | 0.10 |
| Sense of safety (*N* = 106) | -0.34*** | -0.30** | -0.29** | -0.35*** | -0.20* | -0.29** |
| Self-blame (*N* = 64) | 0.05 | 0.12 | -0.14 | 0.03 | 0.17 | -0.14 |
| Acceptance (*N* = 64) | 0.00 | 0.05 | -0.18 | 0.04 | 0.05 | -0.18 |
| Rumination (*N* = 64) | -0.10 | -0.08 | -0.18 | -0.06 | -0.05 | -0.18 |
| Positive refocusing (*N* = 64) | 0.17 | 0.18 | 0.07 | 0.11 | 0.15 | 0.07 |
| Refocus on planning (*N* = 64) | 0.17 | 0.13 | 0.18 | 0.05 | 0.10 | 0.18 |
| Positive reappraisal (*N* = 64) | 0.21 | 0.18 | 0.17 | 0.15 | 0.14 | 0.17 |
| Putting into perspective (*N* = 64) | 0.18 | 0.22 | 0.03 | 0.17 | 0.19 | 0.03 |
| Catastrophizing (*N* = 64) | 0.02 | 0.09 | -0.20 | 0.05 | 0.10 | -0.20 |
| Blaming others (*N* = 64) | 0.04 | 0.01 | 0.09 | 0.03 | -0.05 | 0.09 |

*N — the number of participants who completed each questionnaire*

**p < 0.05, **p < 0.01, ***p < 0.001*
